# Hepatoid adenocarcinoma of the stomach: a unique subgroup with distinct clinicopathological and molecular features

**DOI:** 10.1007/s10120-019-00965-5

**Published:** 2019-04-15

**Authors:** Yakun Wang, Li Sun, Zhongwu Li, Jing Gao, Sai Ge, Cheng Zhang, Jiajia Yuan, Xicheng Wang, Jian Li, Zhihao Lu, Jifang Gong, Ming Lu, Jun Zhou, Zhi Peng, Lin Shen, Xiaotian Zhang

**Affiliations:** 1 Department of Gastrointestinal Oncology, Key Laboratory of Carcinogenesis and Translational Research (Ministry of Education), Peking University Cancer Hospital and Institute, Fucheng Road 52, Haidian District, Beijing, 100142 China; 2 Department of Pathology, Key Laboratory of Carcinogenesis and Translational Research (Ministry of Education), Peking University Cancer Hospital and Institute, Beijing, 100142 China

**Keywords:** Hepatoid adenocarcinoma of the stomach (HAS), Copy number gain (CNG), Chromosome 20

## Abstract

**Objectives:**

Hepatoid adenocarcinoma of the stomach (HAS) is characterized by histological resemblance to hepatocellular carcinoma and a poor prognosis. The aim of this study is to elucidate the clinicopathological and molecular characteristics of HAS.

**Methods:**

Forty-two patients with HAS who received gastrectomy were enrolled in this study. Based on a panel of 483 cancer-related genes, targeted sequencing of 24 HAS and 22 clinical parameter-matched common gastric cancer (CGC) samples was performed. Prognostic factors for overall survival (OS) and disease-free survival (DFS) were analysed with the Kaplan–Meier method.

**Results:**

The most frequently mutated gene in both HAS and CGC was TP53, with a mutation rate of 30%. Additionally, CEBPA, RPTOR, WISP3, MARK1, and CD3EAP were identified as genes with high-frequency mutations in HAS (10–20%). Copy number gains (CNGs) at 20q11.21-13.12 occurred frequently in HAS, nearly 50% of HAS tumours harboured at least one gene with a CNG at 20q11.21-13.12. This CNG tended to be related to more adverse biobehaviour, including poorer differentiation, greater vascular and nerve invasion, and greater liver metastasis. Pathway enrichment analysis revealed that the HIF-1 signalling pathway and signalling pathways regulating stem cell pluripotency were specifically enriched in HAS. The survival analysis showed that a preoperative serum AFP level ≥ 500 ng/ml was significantly associated with poorer OS (*p* = 0.007) and tended to be associated with poorer DFS (*p* = 0.05).

**Conclusion:**

CNGs at 20q11.21-13.12 happened frequently in HAS and tended to be related to more adverse biobehaviour. The preoperative serum AFP level was a sensitive prognostic biomarker for DFS and OS.

**Electronic supplementary material:**

The online version of this article (10.1007/s10120-019-00965-5) contains supplementary material, which is available to authorized users.

## Background

Hepatoid adenocarcinoma of the stomach (HAS), a unique subtype of gastric cancer, is gaining increasing attention in recent years due to its aggressive behaviour, especially the potential for liver metastasis and poor prognosis [1–3]. HAS is characterized by a histological resemblance to hepatocellular carcinoma (HCC); in addition to morphological confirmation, HAS can be confirmed by several immunohistochemical markers, such as AFP, GPC-3, SALL4, and Hap-Par 1 [4, 5]. Previous studies have shown that HAS is the most common of the AFP-producing gastric cancers (GCs), and HAS is commonly believed to have more aggressive biobehaviour and poorer prognosis than cancers without HCC-like morphology [6].

Recent studies have demonstrated that the potential underlying mechanism of HAS may be the common embryonic origin of the stomach and liver from the foregut and that HAS may evolve through genetic progression and/or genetic divergence [7–9]. However, the exact molecular mechanism of HAS is very unclear. Although the TCGA Research Network has defined four major genomic subtypes of gastric cancer—Epstein–Barr virus (EBV)-infected tumours, microsatellite instability (MSI) tumours, genomically stable tumours, and chromosomally unstable tumours [10]—HAS cannot be classified as any of these. Furthermore, retrospective studies showed that none of the patients with elevated levels of AFP mRNA included in the TCGA dataset can be identified as having hepatoid carcinomas due to the lack of HCC-like morphology [11–13], indicating that the HAS subtype is genetically distinct. Like any other carcinoma, HAS is a heterogeneous cancer with different clinical outcomes, biological behaviours, and genetic alterations. Moreover, therapeutic targets specific to this unique subgroup have not been identified.

To better characterize this subset, we analysed 42 gastric adenocarcinomas with at least one focal component resembling HCC differentiation. By using next-generation sequencing (NGS), we aimed to establish a molecular/clinicopathological concept of HAS and to identify new therapeutic targets for this unique cancer.

## Materials and methods

### Case selection and clinicopathological factors

Between 2008 and 2017, 61 patients were diagnosed with HAS at Beijing Cancer Hospital, China. The present study enrolled 42 HAS patients who underwent surgical treatment, including 36 patients with radical resection for gastric cancer and 6 with palliative gastrectomy. Clinical parameters, including age, sex, serum AFP level at diagnosis, primary lesion site, and metastasis status including liver and peritoneal metastasis, were obtained by reviewing the medical records. All tumours were staged according to the TNM staging system of the American Joint Committee on Cancer (7th version, 2009). This study was approved by the ethics committee of Beijing Cancer Hospital. All patients gave written informed consent to allow the use of their tissues for medical research.

### Evaluation of immunohistochemical staining

Pathological diagnosis of HAS was based on the identification of histological features resembling HCC. There was no quantity requirement for the diagnosis of histological hepatoid differentiation; the diagnosis of HAS could also be made for some patients presenting with focal differentiation.

AFP staining was evaluated by two pathologists based on the percentage of stained cells and the staining density. The scores for the percentage of stained cells were classified into three groups: 0 for no stained cells, 1 for 1–50% stained cells, and 2 for 51–100% stained cells. The staining density was scored from 0 to 3: 0 for no staining, 1 for slight staining, 2 for moderate staining, and 3 for intense staining. The two scores were multiplied, resulting in the final stratification of groups by AFP immunohistochemistry (IHC) score: the 1–3 points group and the 4–6 points group.

Ten haematoxylin and eosin-stained slides for every patient were examined by two pathologists to confirm the HAS cell component percentage. Tumours were classified according to HAS cell component percentage with a cutoff value of 50%. In addition, the following histological features were also recorded: tumour size, tumour invasion depth (T stage), lymphovascular invasion, and nodal metastasis. The histological type was determined according to the Lauren classification.

### DNA extraction and NGS

#### Sample collection

All resected specimens were subjected to a uniform preparation protocol for formalin-fixed, paraffin-embedded (FFPE) specimens. Tumour and corresponding nontumour samples were collected from each patient.

DNA was extracted using a QIAamp DNA FFPE Kit (Qiagen, Hilden, Germany) according to the manufacturer’s instructions. The quantification of genomic DNA samples was assessed with a Nanodrop 2000 spectrophotometer (Thermo Fisher Scientific, Inc., Wilmington, DE, USA).

DNA was stored at − 20 °C for future use. At least 500 ng of DNA was required to perform the NGS library preparation. The library was prepared by using KAPA Biosystems library preparation reagents. The library had an average fragment size of 140–200 bp. Hybrid capture was performed by using NimbleGen Capture reagents.

#### Sequencing and data processing

Two fastq files were generated per sample, corresponding to full-length forward and reverse reads. The sequenced raw data were subjected to quality control, including assessment of the base sequence quality, sequence content, GC content and sequence length distribution, and relative percentages of unmatched indices.

The reads were aligned to the hg19 b37 version of the human genome. When calling mutations, an average sequence coverage of ≥ 1000 × was required. Single-nucleotide variant (SNV) and indel calls were subjected to a series of filtering steps to ensure that only high-confidence calls were admitted to the final manual review step. Mutations were annotated by using Annovar software. The somatic MSI status was inferred by interrogating all available genomic microsatellites covered by the 483-gene panel within tumour samples and comparing them against those in the matched normal sample DNA using the MSIsensor program. The specific genes included in the 483-gene panel are listed in Supplementary Table 1.

### Survival analysis and statistical analysis

All patients were regularly followed up from the date of first hospitalization at our centre. The final follow-up date was November 1, 2018. Relapse was defined as local recurrence or distant metastasis. The disease-free survival (DFS) time was calculated from the date of radical surgery to the date of relapse. The overall survival (OS) time was calculated from the date of diagnosis to the last day of follow-up or the date of death.

SPSS 21.0 software was used for statistical analysis. Pearson’s Chi-square test was used to assess the differences between variables. Fisher’s exact test was used when the values were less than five. Survival durations were calculated using the Kaplan–Meier method. For all tests, a *P* value of < 0.05 was considered significant.

## Results

### Clinicopathological findings

A total of 42 patients with HAS who received gastrectomy were evaluated (age 41–78 years, median age 62 years), 36 of whom underwent radical operation and 6 of whom received palliative resection. Most (90.5%) of the HAS patients were male.

AFP is considered the most representative marker of HAS. In our study, serum AFP levels were elevated (> 7 ng/ml) in 20 of the 24 patients. The median AFP level at the time of diagnosis was 236 ng/ml (range 5.3–7335 ng/ml). Of the 24 patients, 10 (41.7%) had a serum AFP level of ≥ 500 ng/ml.

Regarding the primary lesion site, 12 (28.6%) tumours were located at the gastroesophageal junction (GEJ), 5 (11.9%) were located in the gastric body, and 24 (57.1%) were located in the gastric antrum. The majority of HAS (82.1%) tumours coexisted with poorly differentiated adenocarcinoma, whereas only 17.9% coexisted with well-differentiated or moderately differentiated adenocarcinoma.

Of the patients, 4 (9.8%) patients exhibited stage I disease; 12 (29.3%), stage II disease; 19 (46.3%), stage III disease; and 6 (14.6%), stage IV disease.

Regarding the Lauren classification, 25 (71.4%) tumours were classified as intestinal type, 3 (8.6%) were diffuse type, and 7 (20.0%) were mixed type.

Concerning the recurrence patterns, the rate of liver metastasis was as high as 44.4% in the present study population, as expected, whereas only 5.6% of the cases were complicated with peritoneal metastasis. Supplementary Table 2 summarizes the patients’ characteristics.

### Correlation analysis of HAS cell component percentage and serum AFP level, and AFP immunohistochemical staining

**Fig. 1:**
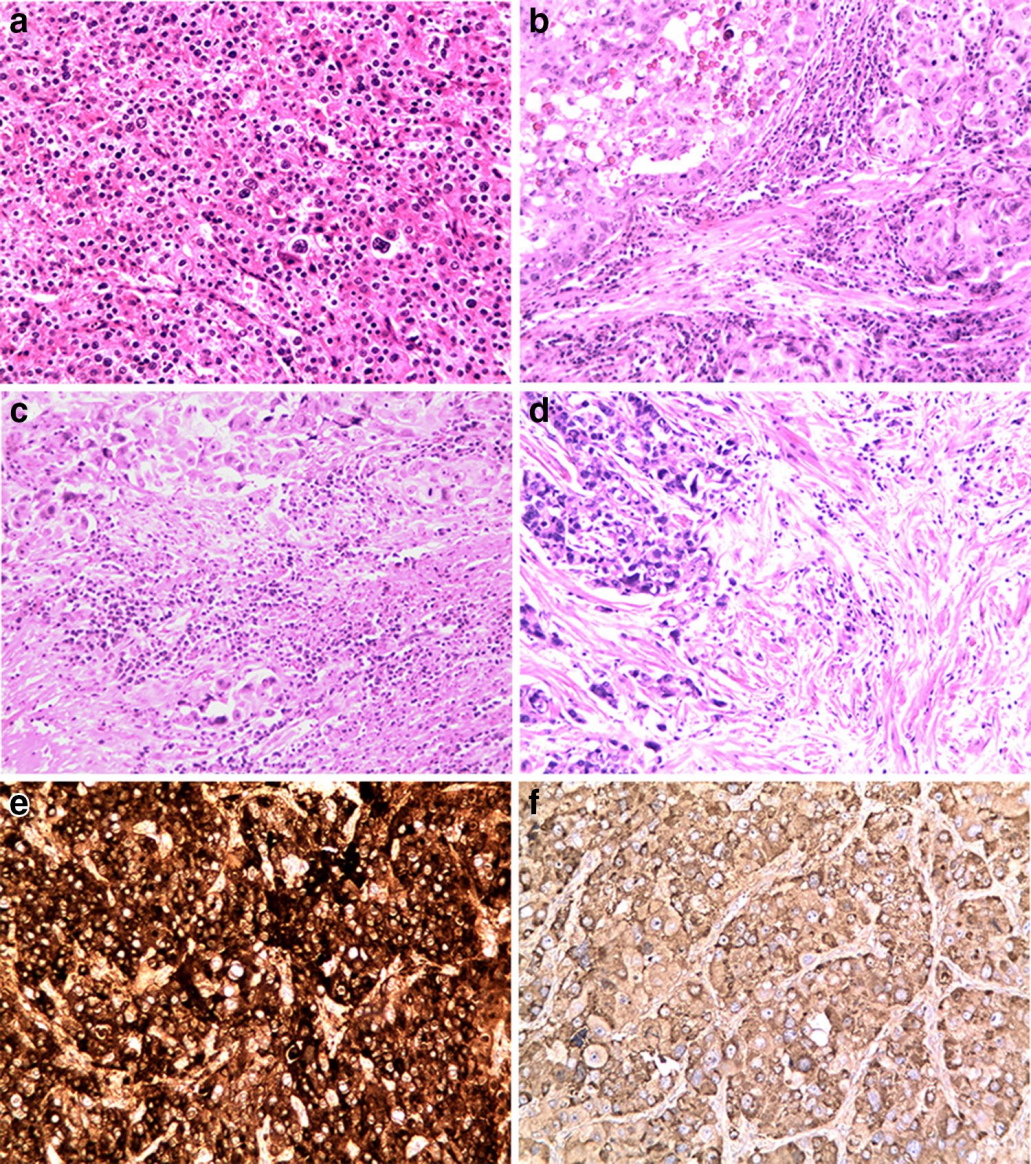
HAS samples with different HAS cell component percentages and different AFP immunohistochemical reactivity scores. **a** 100% HAS cells; **b** 50% HAS cells, with a classic rich cytoplasmic glycogen content and transparent bodies; **c** 25% HAS cells; **d** 10% HAS cells;** e **strong tumour staining;** f **weak tumour staining

Tumours were classified according to the HAS cell component percentage: the cutoff value was 50%, and the percentage of the HCC-like differentiation component varied from 5 to 100%, with a median value of 35%. The AFP IHC results were also classified into two groups: 1–3 points and 4–6 points (Fig. 1a–f).

**Fig. 2:**
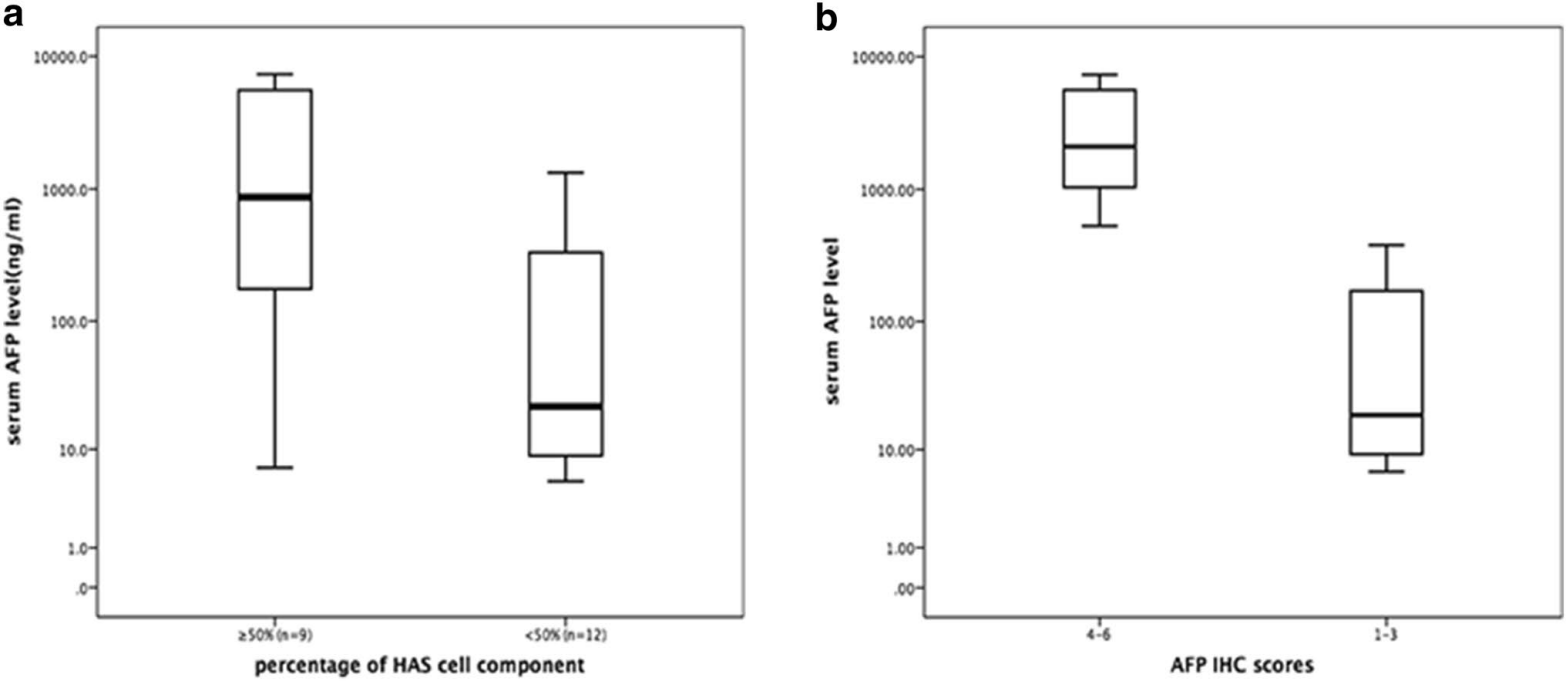
**a** The median serum AFP level of patients with HAS with HAS cell percentages ≥ 50% and < 50% (763.0 and 22.0 ng/ml, respectively, *p* = 0.003). **b** The median serum AFP level of patients with HAS with IHC scores of 1–3 and 4–6 (19.2 and 2316.0 ng/ml, respectively, *p* < 0.001)

We further analysed the relationship between the HAS cell percentage, serum AFP level and AFP immunohistochemical staining score. Fisher’s exact analysis showed that the HAS cell component percentage was associated with the serum AFP level and AFP IHC score, but only the association with the serum AFP level was statistically significant (*p* = 0.003). Moreover, the serum AFP level was in good accordance with the AFP IHC score (Fig. 2a, b). In addition, we found that SALL4, a novel stem cell biomarker, often showed strong positivity in HAS.

### Mutational analysis

From the 42 HAS patients, 24 patients with histologically typical cases were enrolled for NGS. Additionally, we randomly chose 22 clinical parameter-matched patients with common gastric adenocarcinoma (common gastric cancer; CGC) for NGS. The genetic alterations identified are summarized in supplementary Table 3.

We next aimed to investigate the genomic differences between HAS and CGC patients sharing similar clinicopathologic parameters. Among the 24 HAS and 22 CGC sample sequencing results, 1 CGC sample was detected as microsatellite instability high (MSI-H) and 1 HAS and 3 CGC samples had insufficient DNA for analysis, leaving 23 and 18 patients in the HAS and CGC groups, respectively, for NGS analysis. The matched clinicopathological parameters of the two groups are summarized in Supplementary Table 4. Normal tissue adjacent to the tumour (NAT) is commonly used as a control in cancer studies [14, 15], so we did so in our study. After filtering shared mutations found in both tumour tissue and NAT, we identified 167 mutations in 94 genes and 168 mutations in 101 genes in HAS and CGC tumour tissues, respectively.

The most frequently mutated gene in both HAS and CGC tumour samples was TP53, consistent with previous reports and the TCGA database [10, 16]. A total of 7 of the 23 (30.4%) HAS patients and 6 of the 18 (33.3%) CGC patients harboured TP53 mutations. In addition to TP53, CEBPA, RPTOR, WISP3, MARK1, and CD3EAP were detected as high-frequency mutations in HAS (10–20%). In contrast, NF2, C8orf34, NKX2-1, GPR124, FANCA, EPHA7, and CCND2 mutations were detected more frequently in CGC (10–20%). The frequencies of the mutation sites are listed in Table 1.

**Table 1:** High-frequency mutations identified by NGS in HAS

Gene	Frequency in HAS (*n* = 24)	Frequency in TCGA	Case	Mutation sites in HAS
TP53	7 (30.4%)	48.0%	3	c.675_677del:p.225_226del
			6	c.G679C:p.G227R
			8	c.G898T:p.E300X
			9	c.647_649del:p.216_217del
			9	c.647delT:p.I216 fs
			14	c.G703T:p.V235F
			18	c.443-1G > A
			19	c.C551T:p.P184 L
CEBPA	5 (21.7%)	0%	3	c.311_313del:p.104_105del
			15	c.563_564insCTC:p.P188delinsPS
			16	c.311_313del:p.104_105del
			17	c.311_313del:p.104_105del
			22	c.311_313del:p.104_105del
RPTOR	3 (13.0%)	4.0%	9	c.A2272T:p.N758Y
			16	c.G2780A:p.R927Q
			19	c.C1706T:p.P569 L
WISP3	2 (8.7%)	0.7%	9	c.T491A:p.V164E
			22	c.C181T:p.Q61X
MARK1	2 (8.7%)	2.1%	11	c.G131A:p.R44 K
			23	c.G1714A:p.G572S
CD3EAP	2 (8.7%)	2.4%	8	c.G522C:p.K174 N
			10	c.C833T:p.P278L

### Copy number variation analysis

In addition to gene mutation, copy number variation (CNV) has been shown to be associated with the risk and prognosis of different cancers. Since gene amplification is an important mechanism of carcinogenesis, providing a means for the overexpression of cancer-promoting driver genes, CNV was analysed in 23 HAS and 18 CGC patients in this study. CNV analysis was performed using the event-wise testing algorithm based on the read depth of coverage, according to a previous report [17], and samples with copy numbers higher than 3.2 were considered to exhibit copy number gains (CNGs) and were used for further analysis.

CNGs tended to occur more commonly in HAS than in CGC. In HAS patients, we detected 136 CNG events among 86 different genes (fold change 3.22–8.98). However, in the control CGC tumour tissues, a mere 26 CNGs among 19 different genes were detected (fold change 3.2–11.84), demonstrating that HAS is a tumour with significantly increased CNGs.

The genes most frequently showing CNGs in HAS tumour tissues were TOP1 (50.0%), STK4 (45.5%), CDKN1B (40.9%), H3F3A (36.4%), MYC (22.7%), CCNE1 (22.7%), NFKBIA (22.7%), VEGFA (18.2%), CCND3 (13.6%), and E2F1 (13.6%). However, in the CGC tumour tissues, the genes most frequently showing CNGs were CDKN1B (42.9%), H3F3A (23.8%), CDK2 (14.3%), NFKBIA (14.3%), and VEGFA (14.3%).

Notably, a subset of patients with HAS was found to have increased CNGs at the 20q locus; a total of 11 HAS cases (nearly 50%) were found to harbour at least one CNG in genes located at 20q11.21-13.12, while no such changes were detected in CGC tumour tissues (Table 2).

**Table 2:** CNGs in HAS and CGC tumour tissues

Chr	Gene	Start	End	Location	HAS	CGC
1	MAPKAPK2	206,858,607	206,906,131	1q32.1	2 (8.7%)	0
5	SKP2	36,152,269	36,184,167	5q13.2	2 (8.7%)	0
5	RICTOR	38,942,343	39,074,534	5p13.1	2 (8.7%)	0
5	PRKAA1	40,762,862	40,798,318	5p13.1	2 (8.7%)	0
6	VEGFA	43,738,337	43,752,308	6p21.1	6 (26.1%)	2 (11.1%)
11	GSTP1	67,351,215	67,354,073	11q13.2	2 (8.7%)	0
19	CCNE1	30,303,540	30,314,742	19q12	5 (21.7%)	1 (5.6%)
20	ASXL1	30,947,042	31,025,167	20q11.21	2 (8.7%)	0
20	TPX2	30,345,213	30,388,947	20q11.21	2 (8.7%)	0
20	E2F1	32,264,522	32,274,027	20q11.22	3 (13.0%)	0
20	SRC	36,012,542	36,031,797	20q11.23	2 (8.7%)	0
20	TOP1	39,657,979	39,752,005	20q12	11 (47.8%)	0
20	STK4	43,595,147	43,703,877	20q13.12	10 (43.5%)	0
20	NCOA3	46,250,933	46,282,315	20q13.12	2 (8.7%)	0
20	SRMS	62,172,142	62,178,838	20q13.33	2 (8.7%)	0
20	PTK6	62,160,863	62,168,692	20q13.33	2 (8.7%)	0
20	ARFRP1	62,331,739	62,338,496	20q13.33	2 (8.7%)	0
20	FKBP1A	1,350,615	1,373,612	20q13	2 (8.7%)	0
17	ERBB2	37,855,747	37,884,319	17q12	1 (4.3%)	3 (16.7%)
17	CDK12	37,618,308	37,687,592	17q12	0 (0.0%)	3 (16.7%)
12	ERBB3	56,474,026	56,495,855	12q13.2	0 (0.0%)	2 (5.6%)

### Pathway enrichment analysis

**Fig. 3:**
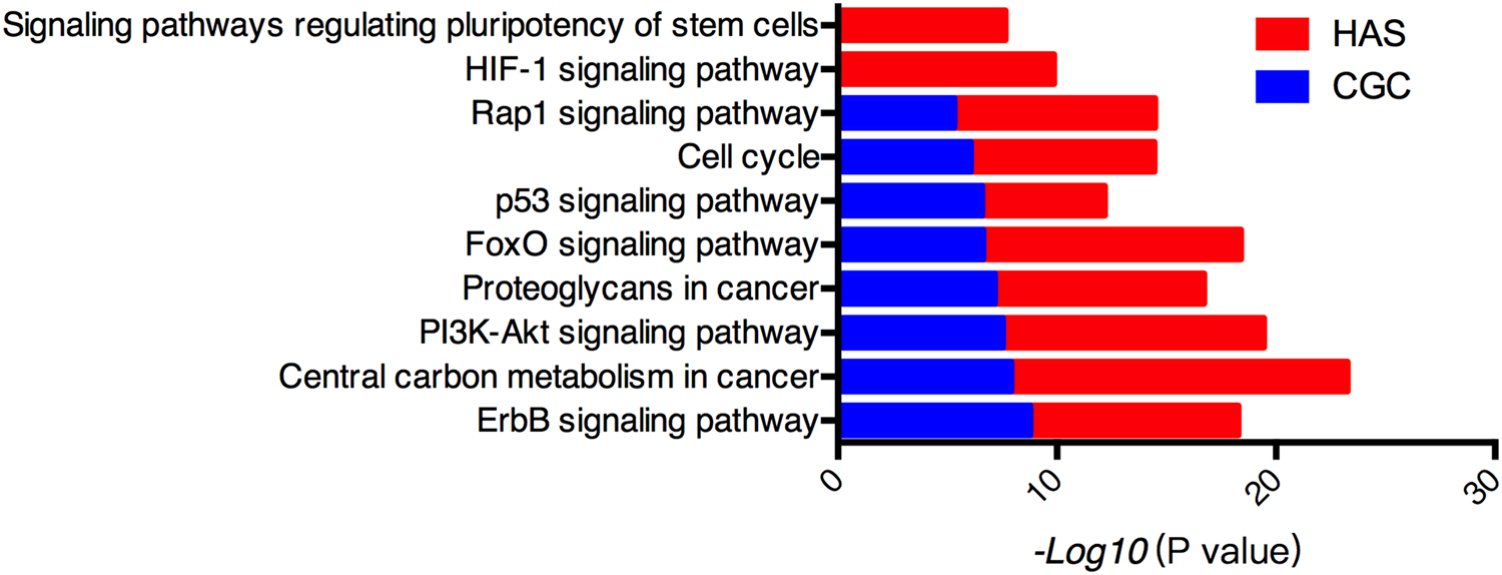
Enriched pathways in HAS and CGC tumour tissues

Furthermore, we mapped genetic alterations in HAS and CGC tumour tissues, including mutations and CNGs, to different pathways and found that several pathways were significantly enriched in the altered genes (Fig. 3). The ErbB signalling pathway, PI3K-Akt signalling pathway, and p53 pathway were the shared enriched altered pathways in both HAS and CGC. In addition, we found that the HIF-1 signalling pathway and signalling pathways regulating the pluripotency of stem cells were specifically enriched in HAS. Enriched pathways in HAS and CGC are listed in Supplementary Table 5.

### Association between CNGs at 20q11.21-13.12 and clinicopathological parameters

Since CNGs at 20q11.21-13.12 were the most frequent genetic alteration in HAS, to further investigate the clinical relevance of this alteration, we next analysed the association between CNGs at 20q11.21-13.12 and the clinicopathological parameters of patients with HAS. In the HAS cohort, CNGs at 20q11.21-13.12 were observed in half of the patients, and we found that the tumours of patients with CNGs at 20q11.21-13.12 might be more aggressive than nonamplified tumours, including poorer differentiation, greater vascular and nerve invasion, and greater liver metastasis, although these differences were not statistically significant (Table 3).

**Table 3:** Associations between CNGs at 20q11.12-13.21 and clinicopathological factors in HAS

Parameters	With 20q11.12-13.21 CNG	Without 20q11.12-13.21 CNG	*P* value
Sex			
Male	9 (90.0%)	9 (81.8%)	0.538
Female	1 (10.0%)	2 (18.2%)	
Age (years)			
≥ 60	5 (50.0%)	6 (54.5%)	0.590
<60	5 (50.0%)	5 (45.5%)	
Primary lesion site			
GEJ	4 (40.0%)	3 (30.0%)	0.500
Non-GEJ	6 (60.0%)	7 (70.0%)	
Serum AFP level			
>500 ng/ml	7 (77.8%)	3/6 (50.0%)	0.287
< 500 ng/ml	2 (22.2%)	3 (50.0%)	
HAS cell component			
≥ 50%	7 (70.0%)	6 (54.5%)	0.392
< 50%	3 (30.0%)	5 (45.5%)	
Lauren subtype			
Intestinal	7 (70.0%)	8 (80.0%)	0.500
Diffuse	3 (30.0%)	2 (20.0%)	
Pathological stage			
1-2	4 (40.0%)	5 (45.5%)	0.575
3-4	6 (60.0%)	6 (54.5%)	
Vascular tumour thrombus			
Yes	10 (100%)	8 (72.7%)	0.124
No	0 (0%)	3 (27.3%)	
Nerve infiltration			
Yes	7 (87.5%)	6 (60.0%)	0.225
No	1 (12.5%)	4 (40.0%)	
Liver metastasis			
Yes	3 (33.3%)	2 (20.0%)	0.444
No	6 (66.7%)	8 (80.0%)	

In addition, patients with CNGs at 20q11.21-13.12 had a trend of higher serum AFP levels and a higher HAS component percentage, although the difference was not statistically significant.

### HAS prognosis

The final follow-up date was November 1, 2018. As of the final follow-up, 16 patients experienced relapse and 20 patients were relapse free. Of the 16 relapsed patients, 13 (81.3%) exhibited recurrence within 1 year after radical gastrectomy; the median duration to recurrence was 6.67 months (1.0–30.7 months), and the median follow-up period of the relapse-free patients was 44.6 months (16.8–120.9 months).

Notably, patients with HAS were very prone to develop liver metastasis; as expected, 72.7% of the relapsed patients in this study had liver metastasis.

**Fig. 4:**
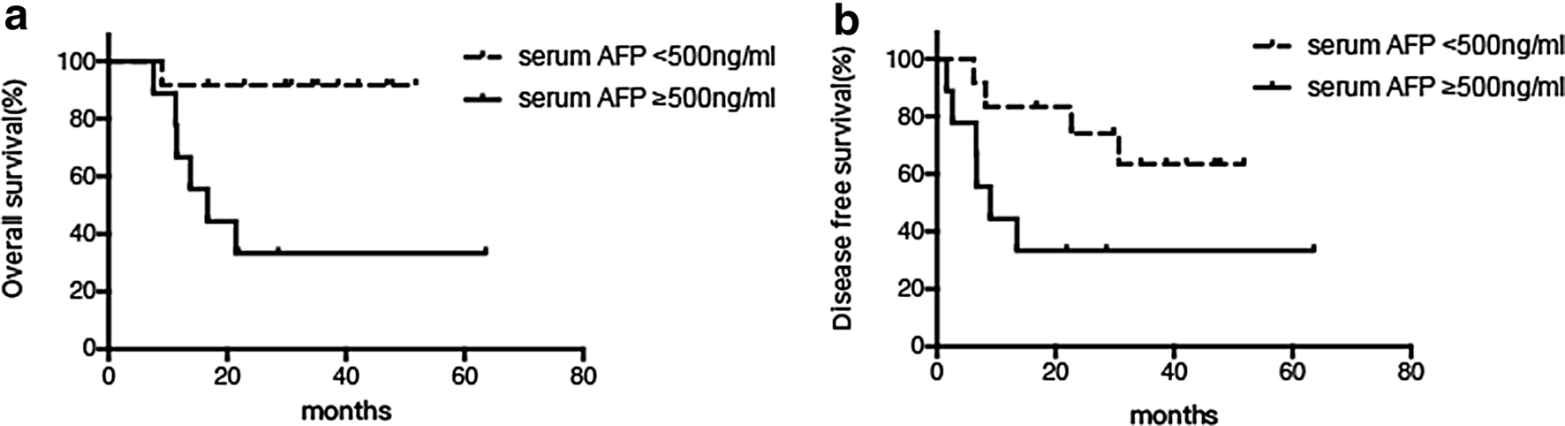
Kaplan–Meier survival plots. **a** The preoperative serum AFP level was associated with OS, *p* = 0.007. **b** The preoperative serum AFP level tended to be associated with DFS, *p* = 0.05

The survival analysis revealed that a preoperative serum AFP level of ≥ 500 ng/ml was significantly associated with poorer OS (*p* = 0.007) and tended to be associated with poorer DFS (*p* = 0.05, Fig. 4a-b).

No significant difference was observed regarding sex, age, or primary lesion site in the survival analysis. Moreover, CNGs at 20q11.21-13.21 were not associated with survival.

## Discussion

Since Ishikura et al. first proposed the new entity HAS in 1985 [18], sporadic cases of this cancer have been reported [19]. HAS is characterized by HCC-like differentiation and is associated with a high incidence of liver metastasis and a poor prognosis.

Similar to the results of previous research [3], this study showed that HAS was characterized by older patient age, male patient sex, intestinal type, and high liver metastasis frequency.

A few studies have examined the histogenesis or development of this cancer and found that the hepatoid cell component was observed only in invasive lesions, indicating that HAS developed from CGC in the mucosa and differentiated into HAS during the process of tumour invasion and proliferation, acquiring the ability to produce AFP [7]. Our result was consistent with this hypothesis, by revealing that the higher the HAS cell component percentage in a tumour, the more is the AFP secreted by the tumour.

Recent attempts have been made to apply NGS technologies to clinical practice, developing innovative precision treatment for different subtypes of gastric cancer. Several comprehensive datasets have revealed many genetic characteristics of gastric cancer [10, 20, 21]. The assemblers of one of these datasets, the TCGA Research Network, defined four major genomic subtypes of gastric cancer: EBV-infected tumours, MSI tumours, genomically stable tumours, and chromosomally unstable tumours. However, retrospective analysis of the TCGA dataset did not provide any information about genetic alterations in HAS, due to its rarity and geographical distribution [11]. The vast majority of HAS case reports are from Asian regions, especially Japan and China. A previous report by Akazawa et al. revealed that HAS may be subcategorized as a solid type of gastric adenocarcinoma with enteroblastic differentiation (GAED) using NGS for 24 patients with GAED, including 3 HAS cases. The most obvious molecular feature of GAED has been reported to be high-frequency TP53 mutations and CNG of ERBB2 [16]. However, our results identified a TP53 mutation rate of 30% in HAS, which is significantly lower than that in GAED (79.2%) and conventional GC (48.0% in the TCGA database) [10, 16], but similar to that in another report by Akiyama in which 33% (5/15) of patients with HAS had a TP53 mutation [7]. In addition, CNG of ERBB2 was not frequently detected in our study. All these differences indicate that HAS may be a genetically distinct subgroup compared with GAED, although many overlapping clinicopathological features exist between the two subcategories of gastric cancer, including a solid pattern and AFP expression.

Considering the existing literature contains very limited information on the molecular features of HAS, we performed NGS with a 483-gene panel in 24 HAS cases and found that DNA CNG at 20q11.21-13.12, which was not detected in CGC, was clearly the most frequent genetic alteration in our HAS cohort. Previous research suggested that 20q amplification might serve as a cancer-initiating event in the development of many cancers [22, 23]. In GC, CNGs at 20q11-13 were detected in 20–30% of cases [24]; moreover, CNVs tended to be more common in intestinal-type cancers than in diffuse-type cancers [25]. In our HAS cohort, the rate of CNG at 20q12.21-13.12 was as high as 50%. More importantly, with respect to associations between clinicopathological characteristics and genetic alterations, CNGs at 20q 11.21-13.12 were associated with more adverse tumour biobehaviour. Therefore, we hypothesize that potential driver genes located at 20q11.21-13.12 may contribute greatly to the carcinogenesis and development of HAS. Among these amplified genes, TOP1 was found to have the highest amplification frequency. In colorectal cancer, the rate of TOP1 CNG has been reported to range from 53 to 84% [26, 27]; moreover, in a metastatic setting, a borderline significant association (*p* = 0.007) between an increase in the TOP1 CN and an objective response to second-line treatment with irinotecan monotherapy has been reported [28]. Although no relevant reports have been presented in previous GC studies, in our study the high frequency of TOP1 CNG in HAS, but not in CGC, suggested that this alteration may become a useful predictive biomarker for TOP1-targeting therapy.

Another interesting observation to note is that SALL4, a novel stem cell gene, is located at 20q13.2. A member of the spalt-like (SALL) gene family (SALL1 to SALL4), SALL4, is a key factor in the maintenance of embryonic stem cell pluripotency and self-renewal [29, 30]. An oncofoetal protein similar to AFP, SALL4 is highly expressed in both the murine and human foetal liver, and its expression declines gradually during development and is silenced in adulthood. SALL4 re-expression is recognized in various cancers and is considered an adverse prognostic factor in HCC, breast cancer, and lung cancer [31, 32]. In gastric cancer, SALL4 was reported to play oncogenic roles through the modulation of epithelial–mesenchymal transition (EMT) and cell stemness [33]. In our study, SALL4 expression was detected in 94.7% and 10.5% of HAS and CGC samples, respectively, similar to the 89.0% vs 15.0% noted in a previous report [5]. In addition, pathway enrichment analysis showed that signalling pathways regulating the pluripotency of stem cells were specifically enriched in HAS. Combining all these results, we speculate that SALL4 may play an essential role in HAS carcinogenesis and that it can be considered a novel target for HAS diagnosis and therapy. Given the oncogenic role of SALL4 and its specific expression in a subset of cancers, its usefulness as a therapeutic target has been explored. In HCC cell lines, the SALL4 expression status was associated with histone deacetylase activity, and treatment with a histone deacetylase (HDAC) inhibitor successfully suppressed the proliferation of SALL4-positive HCC cells [34]. In addition, SALL4-expressing lung cancers are sensitive to treatment with the HDAC inhibitor entinostat [35]. Since there is no relevant report on HAS, further investigations on whether SALL4 targeting can treat HAS should be performed.

Overall, our NGS data showed that HAS is highly genetically distinct, as reflected by the frequent CNGs at 20q12.21-13.12. In addition, the mechanism of genomic instability in HAS may be associated with the overexpression of the stem cell marker SALL4. The specific relation between these characteristics needs further research in the future. To conclude, by comprehensively investigating the molecular features of HAS using NGS, several markers may be considered as therapeutic targets in the future.

As a rare, unique type of gastric cancer, HAS has been reported by many authors to exhibit more aggressive biobehaviour and poorer prognosis than CGC. However, the link between AFP and survival has historically been controversial. In H. Katai’s study, for example, the 5-year survival rate was 34%, and survival after surgery was found to not be linked to the preoperative serum AFP level [36]. However, other studies indicated that a high level of serum AFP is an independent prognostic factor in gastric cancer [37]. The 5-year survival rates for patients with gastric cancer with AFP ≤ 20 ng/ml, 20 < AFP ≤ 300 ng/ml, and AFP > 300 ng/ml were 45.8, 17.8, and 0%, respectively [38].

In the present study, the prognosis of HAS patients who received radical surgery was not as poor as previously thought, with a 5-year survival rate of 41.1%. However, the evaluation of more cases and the use of a longer follow-up period are required to confirm these findings and draw accurate conclusions. However, interestingly, a markedly elevated preoperative serum AFP level (≥ 500 ng/ml), but not a higher HAS cell component percentage was significantly associated with poorer DFS and OS in HAS. Indeed, serum AFP, as the most representative biomarker, has been found to play a critical role in the initiation of HCC progenitor/stem cells [39]. Additionally, among GCs, AFP-producing GCs have higher malignant potential (high proliferative activity, weak apoptosis, and rich neovascularization) than AFP-negative GCs; furthermore, interfering with AFP expression reduced cell invasion and metastasis by enhancing anoikis sensitivity [40]. Therefore, we hypothesize that serum AFP not only is involved in the initiation of HAS, but also plays important roles in tumour progression and invasion.

In summary, this study showed that HAS is genetically characterized by CNGs at 20q11.21-13.12. Investigating and targeting potential driver genes at this locus may provide novel personalized therapies for this rare subtype of GC. The serum AFP level is a prognostic biomarker in HAS, which may also be therapeutically exploitable.

## Conclusions

In conclusion, our analysis showed that CNGs at 20q11.21-13.12 happened frequently in HAS and tended to be related to more adverse biobehaviour. In HAS, the HAS cell component percentage is consistent with the serum AFP level. The preoperative serum AFP level was a sensitive prognostic biomarker for DFS and OS.

## Electronic supplementary material

Below is the link to the electronic supplementary material.

Supplementary material 1 (docx 147 kb)

Supplementary material 2 (docx 59 kb)

Supplementary material 3 (xlsx 269 kb)

Supplementary material 4 (docx 61 kb)

Supplementary material 5 (xlsx 95 kb)
