# Supplementary material for: Hepatoid adenocarcinoma of the stomach: a unique subgroup with distinct clinicopathological and molecular features
Source: Gastric Cancer. 2019 Apr 15;22(6):1183–92. doi: 10.1007/s10120-019-00965-5 (PMC6811386; doi:10.1007/s10120-019-00965-5)
Supplement: Supplementary file 1 — Supplementary material 1 (docx 147 kb) [file 10120_2019_965_MOESM1_ESM.docx]

（483panel列表）

| ABCB1 | ABCC1 | ABCC2 | ABCC4 | ABCC6 | ABCG2 | ABL1 |
| --- | --- | --- | --- | --- | --- | --- |
| ACK1/TNK2 | ACVR1B | AKT1 | AKT2 | AKT3 | ALK | AMER1 |
| APC | AR | ARAF | ARFRP1 | ARID1A | ARID1B | ARID2 |
| ASXL1 | ATIC | ATM | ATP7A | ATR | ATRX | AURKA |
| AURKB | AXIN1 | AXL | B2M | BAIAP3 | BAP1 | BARD1 |
| BCL2 | BCL2L2 | BCL6 | BCOR | BCORL1 | BCR | BIRC5 |
| BLK | BLM | BRAF | BRCA1 | BRCA2 | BRIP1 | BRK/PTK6 |
| BSG/CD147 | BTK | C11orf30 | C18orf56 | C8orf34 | CAMK2G | CAMKK2 |
| CARD11 | **CASP8** | CBFB | CBL | CBR1 | CBR3 | CCND1 |
| CCND2 | CCND3 | CCNE1 | CCR4 | CD19 | CD22 | CD274 |
| CD33 | CD38 | CD3EAP | CD52 | CD74 | CD79A | CD79B |
| CDA | CDC73 | CDH1 | CDK1 | CDK12 | CDK2 | CDK4 |
| CDK5 | CDK6 | CDK7 | CDK8 | CDK9 | CDKN1B | CDKN2A |
| CDKN2B | CDKN2C | CEBPA | CHEK1 | CHEK2 | CHST3 | CIC |
| CSNK1A1 | COMT | CREBBP | CRKL | CRLF2 | CSF1R | CSK |
| CTCF | CTLA4 | CTNNA1 | CTNNB1 | CYBA | CYLD | CYP19A1 |
| CYP1A1 | CYP1A2 | CYP1B1 | CYP2A6 | CYP2B6 | CYP2C19 | CYP2C8 |
| CYP2C9 | CYP2D6 | CYP2E1 | CYP3A4 | CYP3A5 | CYP4B1 | DAXX |
| DDR1 | DDR2 | DNMT1 | DNMT3A | DOT1L | DPYD | DSCAM |
| E2F1 | EGF | EGFL7 | EGFR | EGR1 | EMC8 | EML4 |
| ENOSF1 | EP300 | EPH/EPHA1 | EPHA2 | EPHA3 | EPHA4 | EPHA5 |
| EPHA7 | EPHA8 | EPHB1 | EPHB2 | EPHB3 | EPHX1 | ERBB2/HER2 |
| ERBB3 | ERBB4 | ERCC1 | ERCC2 | ERG | ESR1/ER | ETV1 |
| ETV4 | ETV5 | ETV6 | EWSR1 | EZH2 | FAM46C | FANCA |
| FANCC | FANCD2 | FANCE | FANCF | FANCG | FANCL | FBXW7 |
| FCGR3A | FGF10 | FGF14 | FGF19 | FGF23 | FGF3 | FGF4 |
| FGF6 | FGFR1 | FGFR2 | FGFR3 | FGFR4 | FGR | FKBP1A |
| FLT1 | FLT3 | FLT4 | FOXL2 | FRK | FUBP1 | FYN |
| FZD7 | GALNT14 | GATA1 | GATA2 | GATA3 | GCK | GID4 |
| GINS2 | GNA11 | GNA13 | GNAQ | GNAS | GPC3 | GPR124 |
| GRIN2A | GSK3B | GSTM1 | GSTM3 | GSTP1 | GSTT1 | H3F3A |
| HCK | HGF | HIF-1/HIF1A | HIST1H3B | HNF1A | HRAS | HSP90AA1 |
| IDH1 | IDH2 | IGF1 | IGF1R/IGFR | IGF2 | IGF2R | IKBKB |
| IKBKE | IKZF1 | IL7R | INHBA | INSR/IR | IRF4 | IRS2 |
| ITK | JAK1 | JAK2 | JAK3 | JUN | KAT6A | KDM5A |
| KDM5C | KDM6A | KDR/VEGFR | KEAP1 | KIT | KLC3 | KLHL6 |
| KMT2A/MLL | KMT2B/MLL4 | KMT2C/MLL3 | KMT2D/MLL2 | KRAS | LCK | LIMK1 |
| LMO1 | LRP1B | LRP2 | LYN | MAP2K1 | MAP2K2 | MAP2K4 |
| MAP3K1 | MAP4K4 | MAP4K5 | MAPK1 | MAPK10 | MAPK14 | MAPK8 |
| MAPK9 | MAPKAPK2 | MARK1 | MCL1 | MDM2 | MDM4 | MED12 |
| MEF2B | MEN1 | MERTK | MET | MITF | MKNK2 | MLH1 |
| MPL | MRE11A | MS4A1 | MSH2 | MSH6 | MTDH | MTHFR |
| MTOR | MTRR | MUTYH | MYC | MYCL1 | MYCN | MYD88 |
| NAT1 | NAT2 | NCAM1 | NCF4 | NCOA3 | NCOR1 | NEK11 |
| NF1 | NF2 | NFE2L2 | NFKBIA | NKX2-1 | NOS3 | NOTCH1 |
| NOTCH2 | NPM1 | NQO1 | NRAS | NTRK1 | NTRK2 | NTRK3 |
| NUP93 | PAK1 | PAK3 | PALB2 | PARP1 | PARP2 | PAX5 |
| PBRM1 | PDCD1 | PDGFRA | PDGFRB | PDK1 | PHF6 | PHKA2 |
| PIGF | PIK3CA | PIK3CB | PIK3CG | PIK3R1 | PIK3R2 | PKC/PRRT2 |
| PKCγ/PRKCG | PKCε/PRKCE | PLK1 | PPARD | PPP1R13L | PPP2R1A | PRDM1 |
| PRDX4 | PRKAA1 | PRKAR1A | PRKCA | PRKCB | PRKDC | PTCH1 |
| PTEN | PTK2 | PTPN11 | PTPRD | RAC2 | RAD50 | RAD51 |
| RAF1 | RARA | RB1 | RET | RICTOR | RMDN2 | RNF43 |
| ROCK1 | RON/MST1R | ROS1 | RPL13 | RPS6KA1 | RPS6KB1 | RPTOR |
| RRM1 | RUNX1 | SCF/KITLG | SDHA | SDHAF1 | SDHAF2 | SDHB |
| SDHC | SDHD | SETD2 | SF3B1 | SGK1 | SHH | SIK1 |
| SKP2 | SLC10A2 | SLC15A2 | SLC22A1 | SLC22A16 | SLC22A2 | SLC22A6 |
| SLCO1B1 | SLCO1B3 | SMAD2 | SMAD4 | SMARCA4 | SMARCB1 | SMO |
| SOCS1 | SOD2 | SOX10 | SOX2 | SOX9 | SPEN | SPG7 |
| SPOP | SRC | SRD5A2 | SRMS | STAG2 | STAT1 | STAT2 |
| STAT3 | STAT4 | STAT5A | STAT5B | STAT6 | STEAP1 | STK11 |
| STK3 | STK4 | SUFU | SULT1A1 | SULT1A2 | SULT1C4 | SYK |
| TCF7L1 | TCF7L2 | TEK | TET2 | TGFBR1 | TGFBR2 | TK1 |
| TMPRSS2 | TNF | TNFAIP3 | TNFRSF14 | TNFRSF8 | TNFSF11 | TNFSF13B |
| TOP1 | TP53 | TPMT | TPX2 | TRAIL-R1 | TRAIL-R2 | TSC1 |
| TSC2 | TSHR | TYMS/TS | TYRO3 | U2AF1 | UBE2I | UGT1A1 |
| UGT1A9 | UGT2B15 | UGT2B17 | UGT2B7 | UMPS | VEGFA | VEGFB |
| VHL | WEE1 | WISP3 | WNK3 | WT1 | XPC | XPO1 |
| XRCC1 | XRCC4 | YES1 | ZAP70 | ZC3HAV1 | ZNF217 | ZNF703 |
| ALK | AML1 | BCR | BRAF | EGFR | ETV1 | ETV4 |
| ETV5 | ETV6 | EWSR1 | MLL | MYC | NTRK1 | PDGFRA |
| RAF1 | RARA | RET | ROS1 | TMPRSS2 |  |  |
