# Supplementary material for: Hepatoid adenocarcinoma of the stomach: a unique subgroup with distinct clinicopathological and molecular features
Source: Gastric Cancer. 2019 Apr 15;22(6):1183–92. doi: 10.1007/s10120-019-00965-5 (PMC6811386; doi:10.1007/s10120-019-00965-5)
Supplement: Supplementary file 2 — Supplementary material 2 (docx 59 kb) [file 10120_2019_965_MOESM2_ESM.docx]

**S Table 2: Clinicopathological features of 42 patients with HAS**

| Characteristics | No. of patients (%) |
| --- | --- |
| Sex  Male  Female | 38 (90.5%)  4 (9.5%) |
| Age (median 62)  ≥60  <60 | 25 (59.5%)  17 (40.5%) |
| Surgery type  Radical  Palliative | 36 (85.7%)  6 (14.3%) |
| Serum AFP level at diagnosis  (median 236 ng/ml)  ≥500  <500 | 10 (41.7%)  14 (58.3%) |
| Primary lesion site  GEJ  Gastric body  Gastric antrum  Gastric remnant | 12 (28.6%)  5 (11.9%)  24 (57.1%)  1 (2.4%) |
| Differentiation degree  Poor  Well | 32 (82.1%)  7 (17.9%) |
| Tumor diameter, maximum  ≥5 cm  <5 cm | 19 (51.4%)  18 (48.6%) |
| Lauren classification  Intestinal  Diffuse  Mixed | 25 (71.4%)  3 (8.6%)  7 (20.0%) |
| Pathological stage  I  II  III  IV | 4 (9.8%)  12 (29.3%)  19 (46.3%)  6 (14.6%) |
| Liver metastasis  Present  Absent | 12 (32.4%)  25 (55.6%) |
| Peritoneal dissemination  Present  Absent | 2 (5.6%)  34 (94.4%) |
