# Supplementary material for: Hepatoid adenocarcinoma of the stomach: a unique subgroup with distinct clinicopathological and molecular features
Source: Gastric Cancer. 2019 Apr 15;22(6):1183–92. doi: 10.1007/s10120-019-00965-5 (PMC6811386; doi:10.1007/s10120-019-00965-5)
Supplement: Supplementary file 4 — Supplementary material 4 (docx 61 kb) [file 10120_2019_965_MOESM4_ESM.docx]

**S Table 4: Comparison of clinicopathological data between HAS and CGC group**

| Clinicopathological  Characteristics | HAS（n=23） | CGC（n=18） | *P* value |
| --- | --- | --- | --- |
| Age  ≥60  <60 | 12(52.2%)  11 | 11(61.1%)  7 | 0.400 |
| Sex  M  F | 20(87.0%)  3 | 17(94.4%)  1 | 0.402 |
| Surgical type  Radical  Palliative | 21(91.3%)  2 | 18(100%)  0 | 0.309 |
| Pre-operative chemo  No  Yes | 16(72.7%)  6 | 16(72.7%)  6 | 0.632 |
| Primary site location  GEJ  Non-GEJ | 8(34.8%)  15 | 6(33.3%)  12 | 0.594 |
| Lauren subtype  Intestinal  Non-intestinal | 16(72.7%)  6 | 12(66.7%)  6 | 0.471 |
| Differentiation degree  Well  Poor | 6(26.1%)  17 | 6(33.3%)  12 | 0.434 |
| Tumor diameter(maximum)  ≥5cm  <5cm | 10(50.0%)  10 | 5(27.8%)  13 | 0.143 |
| pTNM staging  I-II  III-IV | 10(43.5%)  13 | 7(38.9%)  11 | 0.510 |
| Vascular tumor thrombus  Yes  No | 20(87.0%)  3 | 9(50.0%)  9 | 0.012 |
| Nerve infiltration  Yes  No | 15(75.0%)  5 | 6(60.0%)  4 | 0.331 |
| Liver metastasis  Yes  No | 8(36.4%)  14 | 8(57.1%)  6 | 0.190 |
| HER 2  Positive  Negative | 2(9.5%)  19 | 5(33.3%)  10 | 0.089 |
